# Understanding COVID: Collaborative Government Campaign for Citizen Digital Health Literacy in the COVID-19 Pandemic

**DOI:** 10.3390/life13020589

**Published:** 2023-02-20

**Authors:** Mónica López-Ventoso, Marta Pisano González, Cristina Fernández García, Isabel Diez Valcarce, Inés Rey Hidalgo, María Jesús Rodríguez Nachón, Ana María Menéndez García, Michelle Perello, Beatrice Avagnina, Oscar Zanutto, Alberto Lana

**Affiliations:** 1 General Directorate of Care, Humanization and Socio-Health Care, 33005 Oviedo, Asturias, Spain; 2 Health Research Institute of the Principality of Asturias (ISPA), 33006 Oviedo, Asturias, Spain; 3 Foundation for the Promotion in Asturias of Applied Scientific Research and Technology (FICYT), 33007 Oviedo, Asturias, Spain; 4 Consult Europa Projects and Innovation, 35006 Las Palmas de Gran Canaria, Las Palmas, Spain; 5 Institute for Hospitalization and Assistance Services for the Elderly (ISRAA), 31100 Treviso, Veneto, Italy; 6 Department of Preventive Medicine and Public Health, School of Medicine and Health Sciences, University of Oviedo 33003 Oviedo, Asturias, Spain

**Keywords:** eHealth, (d)health literacy, health literacy, health intervention, health strategy, digital health, pandemic, COVID-19

## Abstract

The strategy “Understanding COVID” was a Public Health campaign designed in 2020 and launched in 2021 in Asturias-Spain to provide reliable and comprehensive information oriented to vulnerable populations. The campaign involved groups considered socially vulnerable and/or highly exposed to COVID-19 infection: shopkeepers and hoteliers, worship and religious event participants, school children and their families, and scattered rural populations exposed to the digital divide. The purpose of this article was to describe the design of the “Understanding COVID” strategy and the evaluation of the implementation process. The strategy included the design and use of several educational resources and communication strategies, including some hundred online training sessions based on the published studies and adapted to the language and dissemination approaches, that reached 1056 people of different ages and target groups, an accessible website, an informative video channel, posters and other pedagogical actions in education centers. It required a great coordination effort involving different public and third-sector entities to provide the intended pandemic protection and prevention information at that difficult time. A communication strategy was implemented to achieve different goals: reaching a diverse population and adapting the published studies to different ages and groups, focusing on making it comprehensible and accessible for them. In conclusion, given there is a common and sufficiently important goal, it is possible to achieve effective collaboration between different governmental bodies to develop a coordinated strategy to reach the most vulnerable populations while taking into consideration their different interests and needs.

## 1. Introduction

### 1.1. COVID-19 Pandemic and Vulnerable Populations

A viral outbreak of an unknown coronavirus (SARS-CoV-2) was declared a pandemic by the World Health Organization (WHO) in March 2020. The increasing rate of incidence and mortality from the associated disease (COVID-19) challenged and stressed healthcare institutions and the global economy and had an impact on the physical and mental health of people around the world. The effects of this pandemic forced the adoption of drastic collective prevention measures throughout the world. In Spain, a state of alarm was decreed, followed by a series of agreements and resolutions on preventive measures and recommendations related to SARS-CoV-2 infection [1], which broke into the normal organization of administrations and the population. Collective confinement stands out among all measures adopted and how it profoundly transformed the general way of living and how to act and confront COVID-19.

As the pandemic evolved, the natural history of COVID-19 and its comprehension suffered great advances. From the beginning, it was suspected that the burden of disease between urban and rural areas could be different [2]. According to Lakhani and colleagues, once the virus entered a rural community, there was a higher relative rate of morbidity and mortality [3]. These higher rates would go unnoticed if specific epidemiological monitoring were not carried out in rural areas, given that the population impact is low because they represent a low percentage of the total population [4]. In addition, the rural environment is characterized by inequity in access to health infrastructure, health literacy, preparation and adaptation for the pandemic, greater difficulties in changing social and/or work habits, and demographic aging that conditions high rates of physical frailty, morbidity and dependency [3,5].

Although the characteristics of the Spanish rural environment are not comparable to those of the aforementioned studies, in Spain, there was also concern about the epidemiological vulnerability of the rural population. This situation, together with the analysis presented, justified the need to adapt intervention strategies in the prevention and control of transmission in rural and aged areas. The rural population is the most vulnerable, but other existing vulnerable populations must be included in the prevention strategies as there is strong evidence about the different impacts of COVID-19 on other groups. Making a general health information approach expecting that the resulting information will be acceptable for different groups is not sufficient to achieve equity, and a multifaceted approach that reaches every group is therefore needed.

### 1.2. Decision-Making in the COVID-19 Crisis and Public Health Strategies

During the early phases of the COVID-19 pandemic, healthcare professionals worked under high levels of uncertainty. Soon a pressing need emerged to translate knowledge into practice more efficiently, with rapid assessment and dissemination of scientific evidence to guide decision-making [6]. Some studies found that bringing together experts from academia, science and clinical practice to search for and summarize information of high scientific quality was effective for informed decision-making [7]. However, knowledge was not only needed by clinical and public health decision-makers as the general population also had a compelling information need to make the best choices for their health. In addition, an ‘infodemic’ led to confusion and distrust in health workers weakening public health responses [8].

**Figure 1:**
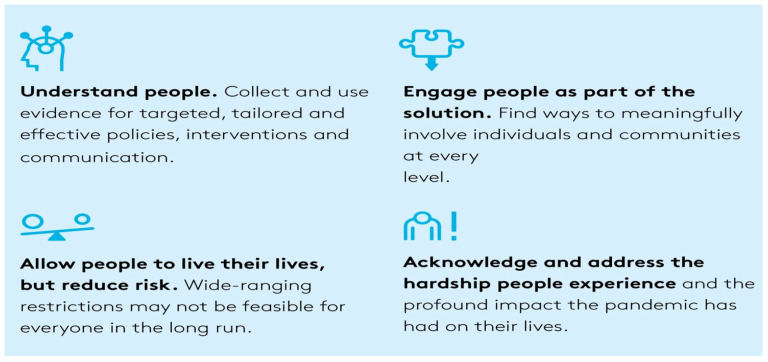
WHO: Pandemic fatigue: proposal of four key strategies for governments to maintain and reinvigorate public support for protective behaviors.

Moreover, the continuous demand for efforts from the population to comply with the requirements and recommendations to improve the epidemiological and health situation (for example, confinements, social distancing, travel restrictions, cuts in benefits, vaccination, etc.) also required efforts to convey information clearly and understandably. However, with the passage of time, the transmission of information and the training of citizens from the public administrations became increasingly complex, a circumstance that especially affected the most vulnerable groups [9], and the first signs of “pandemic fatigue” in the general population began to show. This is why the WHO urged the inclusion of four recommendations in dissemination campaigns and actions summarized in Figure 1 [10].

### 1.3. The “Understanding COVID” Strategy

For all these reasons, in most regions, it became necessary to implement information strategies tailor-made for the general population, especially for the most vulnerable people. “Understanding COVID” was a strategy that began to be developed in March 2020 at the General Directorate of Care, Humanization and Socio-Health Care of Asturias (Spain). Asturias is the most aged region in Spain and one of the most aged in Europe. In addition, although a large part of its population lives in diffuse urban environments [11,12], a large number of small and dispersed rural centers that are difficult to access also exist. Therefore, the rural area of Asturias presents some particular problems leading to a risk of poverty or social exclusion: the demographic situation (shortage of population, exodus of inhabitants and aging of the population in rural areas); the difficulties for the mobility of the population (lack of infrastructures and basic services, lack of adequate transport connections); or problems related to the labor market (lower employment rates and long-term unemployment).

The general objective of the “Understanding COVID” strategy was to increase the offer of digital information on prevention and protection against COVID-19, individual care and emotional approach to both the largest number of citizens, prioritizing vulnerable groups and the rural environment of Asturias. The specific objectives were to:

1. Listen to citizens’ voices to redesign training actions, keeping in mind the suggestions from the community and acknowledging the difficulties and successes in carrying out the recommended protection measures against COVID-19.
2. Search, simplify, and adapt in a more comprehensive way to the community all the information and evidence available to increase people’s protection against COVID-19.
3. Promote accessible information on protection measures for citizens in general and for people with hearing or visual disabilities.
4. Adapt digital health literacy (or (d)HL) to the particular needs of the population groups to which it is directed (adult population, young people, fathers and mothers, etc.).
5. Design specific campaigns for sectors of activity that are particularly exposed, such as workers in poorly ventilated places and/or environments with a large influx of people.
6. Work with children and adolescents to increase safety in the school environment.

The “Understanding COVID” strategy had various target population groups, which included citizens of rural areas, citizens of urban areas, municipal technical professionals, people of Roma ethnic origin, immigrants, and citizens with impaired vision and/or hearing. Another relevant focus to work with was the job sectors most exposed to COVID-19: local commercial activities carried out in indoor areas like hairdressers, beauty salons, and places of worship. In particular, tourism outlets and hotels developed training to protect their own activity, increasing their own security measures and “how to use the facilities” materials for their customers. Finally, in the design of the “Understanding COVID” strategy, two groups were also taken into account in a differentiated way, schoolchildren and also their families, since children and adolescents were left out of the pedagogical approach used for the adult population, and other participation and information methodologies were incorporated.

Given that the “Understanding COVID” strategy was a population-based public health campaign, no sampling method was used, but it was disseminated throughout the target population in order to reach the largest possible number of vulnerable people and later study the scope and effectiveness of the strategy under real conditions, within the broad framework of the implementation science.

### 1.4. Purpose of this Article

Currently, a large number of national and subnational governments are conducting retrospective evaluations of COVID-19 health policy decisions and actions to reflect on their strengths and weaknesses and, thus, to find opportunities to reinforce public institutions for future crises [13]. Thereby, the main aim of this article was to describe the design of the “Understanding COVID” strategy of the Ministry of Health of the Principality of Asturias (Spain), which helped to offer and to adapt information on protection measures against COVID-19 for the entire population of Asturias, including how specific criteria were incorporated for the design of interventions in rural areas with the active participation of their inhabitants. Secondarily, the article also shows the preliminary evaluation of the implementation process of the strategy under real conditions. Ultimately, this work allows us to understand the Spanish public health sector’s capacities to deal with crises and seeks to generate learning toward a more effective and equitable response in the future.

## 2. Design Phases and Preliminary Evaluation of the Implementation Process of the “Understanding COVID” Strategy

### 2.1. Hearing Citizen’s Voice

The first step in designing the strategy was based on analysis through a survey to evaluate weaknesses, perceived strengths, and topics and contents of interest. This was sent by phone using the WhatsApp application, adapting to the technology available at the time of confinement. The survey met the requirements of Organic Law 3/2018 [14] in terms of data analysis and dissemination. Participants were asked to answer six questions on a 6-item Likert scale (0: Strongly disagree; 1: Disagree; 2: Slightly Disagree; 3: Slightly Agree; 4: Agree; 5: Strongly agree) and an additional open-ended question to capture proposals on COVID-19 prevention and control training (“What else would you like us to include in that training? Write down what you deem important in these times of pandemic”).

Table 1 shows the responses to this first online survey incorporating the opinion of citizens between 9 March 2020 and 13 March 2020 (106 responses in one week). The questions were not mandatory, so not all of them were answered by all respondents, and the average response rate was 93%. The percentage of agreement was greater than or equal to 80% in the answer “strongly agree” in questions 1, 3, 5, and 6 and greater than or equal to 65% in the same answer in questions 2 and 4.

In the open-ended question, respondents answered that their greatest interest was the formations focused on the use of “personal protective equipment”, information on face masks and on dealing with emotions in times of pandemics.

The continuous collection of information from participants was considered a priority due to the relevance of incorporating real doubts and needs, as well as adapting the strategy to all target audiences. Thereby, in a later phase, a survey was designed and sent to all participants who took part in the training so that they could anonymously and voluntarily evaluate the usefulness, accessibility, contents, teaching methodology and satisfaction, as shown in Table 2. Along with the previous results, information was also collected from the participants in the training sessions (e.g., opinions or testimonials), thanks to which new content was al-so developed and work was done with groups or sectors that were valued as relevant at different times. 

A total of 472 responses were collected from the continuous information and satisfaction survey. Of these, 65 were from participants in the activity aimed at the school families’ associations, 111 from those aimed at secondary schools, 256 from the “Drop by Drop” action training designed for individual citizens, and 40 from the “First Quality Air”, the specific training offered to the hotel industry.

The percentages of agreement for usefulness, time and accessibility based on the Likert scale are shown in Table 3. In summary, when asked about the usefulness of the training, the activity with the best result was the training for catering “First Quality Air” with 88% “strongly agree” followed by the training of secondary schools with 86%, family associations with 78% and “Gota a Gota” with 77%. In relation to the duration of the training, all four pieces of training have high scores of “yes”, with the secondary schools’ training reaching 100% agreement, followed by the Family Associations’ training with 97% and “Gota a Gota” and “First Quality Air” with 95% each. In the section related to accessibility, the “strongly agree” scores ranged from 86% for training in secondary schools, 83% for Family Associations and “Gota a Gota,” and finally, “First Quality Air” with 80%.

The percentages of agreement with the satisfaction-based question on the Likert scale are shown in Table 4. In this table, we can be seen that satisfaction was measured with a broader Likert scale (0–10), with the highest score in all cases being 10, with 49% in families’ associations, 85% in “First Quality Air “, 86% in secondary schools and finally 89% in the “Drop by Drop”.

Along with the previous results, information was also collected from the participants in the training sessions (e.g., opinions or testimonials), thanks to which new content was also developed and work was done with groups or sectors that were valued as relevant at different times.

### 2.2. Informative Content

In order to prepare the contents of the “Understanding COVID” strategy, the needs expressed by the citizens and identified in the previous phase were taken into consideration. These needs were articulated around four core themes:

- Self-protection and collective protection measures against COVID-19: including frequent hand washing, interpersonal distance and coughing into the cubital fossa, use of masks, cleaning of domestic environments, collective protection measures and specific environments, and the disinfection of physical spaces.
- Identification and containment of the sources of contagion: early diagnosis of people with symptoms, isolation of cases and tracing and quarantine of close contacts. Therefore, it was important to publicize the symptoms and the protocols for reporting them (e.g., health personnel in the area).
- Content related to emotional management: assertiveness, managing emotions in difficult times, such as facing fear, leaving home after confinement, love, learning to trust, positive thinking, guided visualization, etc.
- Contents related to maintaining healthy habits: healthy diet, physical exercise, maintenance of routines, communication and sleep.

Next, personnel trained in documentation and communication conducted a search for all the information available at that time in different sources: scientific literature (PubMed and Web of Science), gray literature, expert information (documents and explanatory videos), documentation of official health agencies and web resources. Finally, the relevant information was analyzed and synthesized by a group of experts and distributed in the communication channels of the “Understanding COVID” strategy: one created the informative content on the web (in text, in video or in infographics) and others for online training. The contents were continuously reviewed to ensure the maximum topicality of the information that was so changing at that time, to adapt it and adapt it to an understandable language for the different target audiences, as well as to align the messages with the policies that were required in terms of prevention and protection by the authority at any given time.

**Figure 2:**
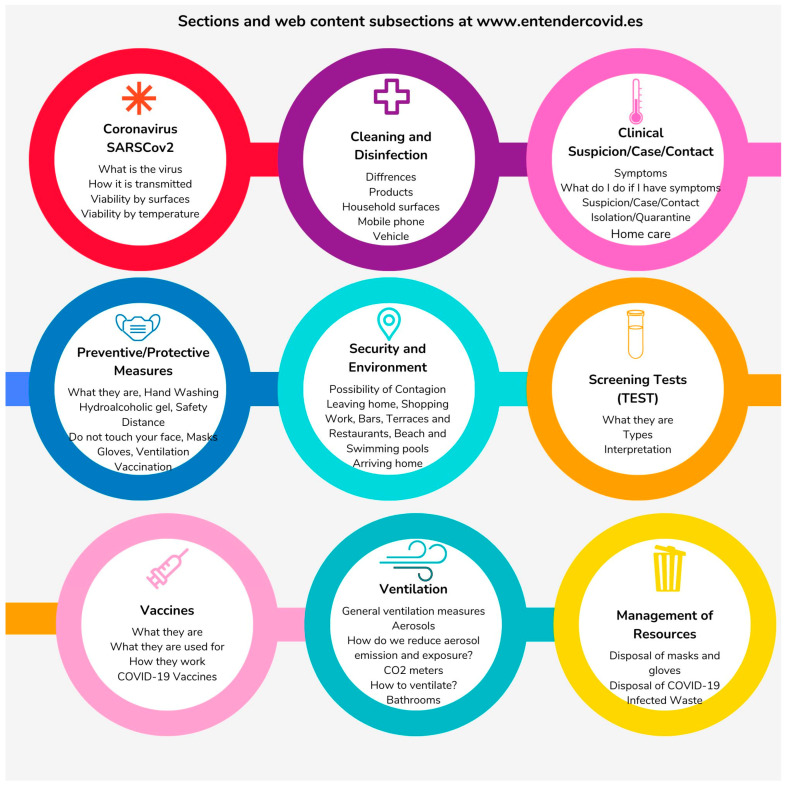
Sections and web content subsections at www.entendercovid.es accessed on 1 February 2023.

As a result, more than 150 documentary sources were consulted. A total of 10 sections containing the most relevant information were grouped together, and 50 subsections of key information (Figure 2), with 55 infographics and videos.

### 2.3. Digital Health Literacy

The central methodological pillar of the “Understanding COVID” strategy was the live training sessions. The sessions were virtual, using the office tool “Microsoft Teams”, which allows access via smartphone (preferred), PC or tablet without the need to install any type of software. The sessions lasted 60–90 min and were structured into two well-differentiated parts: a first part where updated information on the pandemic was presented (30–45 min), and a second part where there was free time for questions from attendees to resolve doubts and explore their needs and barriers to implement protection measures (30–45 min). During the design phase of the training sessions, special emphasis was placed on adapting the content, images and language to vulnerable populations (e.g., residents of rural areas, Roma, caregivers, etc.). In addition, attention was paid to adapting the temporary programming of the sessions to the working hours of the professional groups.

For the development of the online training sessions and the recruitment of attendees, there was a collaboration from 54 municipalities of Asturias (out of a total of 78, or 69.2%) that adhered to the strategy. Likewise, the Ministry of Education, Ministry of Tourism and Sports, associations of the third sector (hotels, patients, neighbors, etc.), associations of families, as well as associations of Roma ethnic. Thanks to this collaborative work, a total of 100 interventions were carried out with an overall attendance of 1056 people. A summary of the population groups reached in the training sessions can be seen in Table 5.

**Figure 3:**
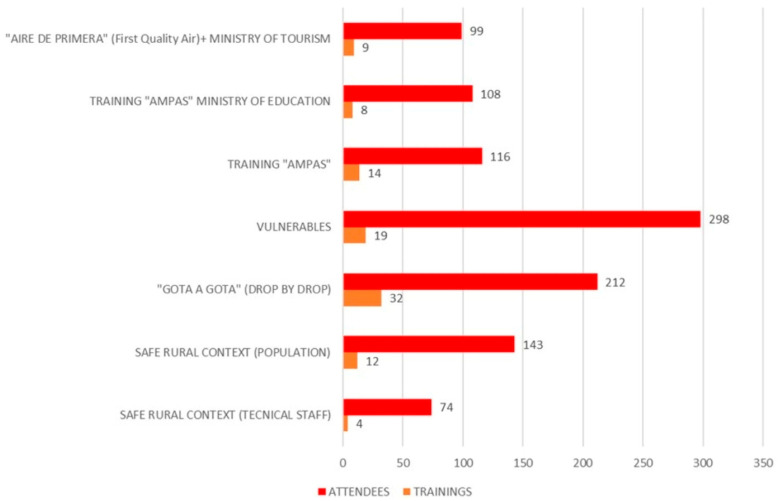
Number of training sessions and participants. AMPAS: Spanish acronym for Families Associations.

Figure 3 depicts the main training actions developed by the strategy, among which the following clearly stand out: “Drop by drop”, that is, general training for individually enrolled citizens, with 32 actions; training for vulnerable populations, with 19 actions; and training for parents of children in confinement and/or isolation, as well as specific training for parents of children with special needs, with 14 actions.

Lastly, in the open-ended questions of the questionnaire, the most repeated messages correspond to the following codes: “gratitude”, “appreciation of the live session for questions and needs”, “request to repeat the training to update knowledge”, and “verification of the need for training for the entire population”.

### 2.4. Communicative Materials and Accessibility of Information

In order to achieve the objectives related to reaching the maximum number of citizens, making adaptations for different groups, achieving accessibility of language and content of materials, breaking the digital divide and making information accessible to citizens with accessibility and equity, the strategy “Understanding COVID” designed, coordinated and produced the following communication materials, which were made available in March 2021 to the target population.

#### 2.4.1. Logo and Graphic Identity

**Figure 4:**
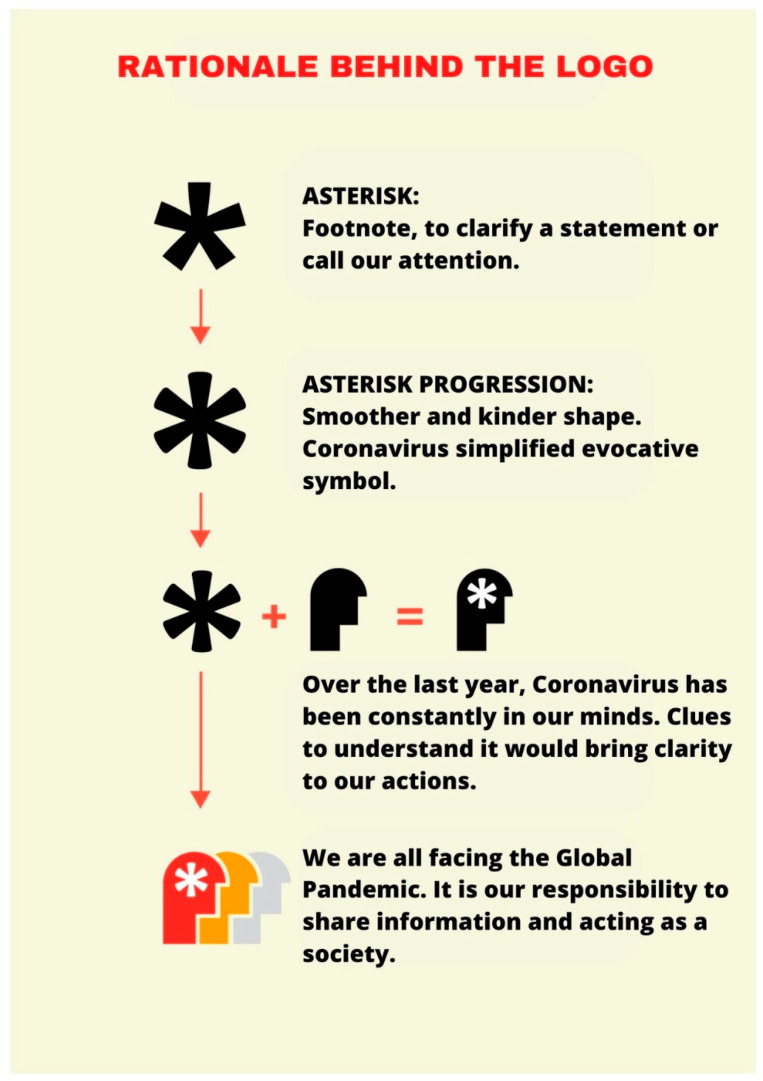
Understanding COVID: logo development.

The design of the logo and the graphic identity of the campaign were part of the methodology of the “Understanding COVID” strategy (Figure 4). Reaching the public, involving them in their health decisions and reflecting on the available evidence were the core of the starting elements for the design of the logo and the graphic identity of the strategy.

#### 2.4.2. Web Page

An independent web page (www.entendercovid.es, accessed on 1 February 2023) with free and open access was developed, which provided access to all the informative material of the strategy, including documents, ad hoc infographics and videos. The web page also had an interactive virtual space for solving doubts, as well as suggestions and actions for the campaign. The accessibility of the page was reviewed from its design by the Spanish National Organization for the Blind to guarantee inclusiveness. In addition, the videos were designed to include sign language for the deaf community. The following principles were taken into account when designing the website:

- Didactic vocation: Present the information in an orderly, clear and attractive way. Carry out positive communication avoiding contributing to general pandemic fatigue.
- Usability: Simple and intuitive navigation. Increase click efficiency (relevant information in the minimum number of clicks). Prioritize information in plain text.
- Accessibility: Information intended for the whole of society. Visual codes are understandable by all. Its simple structure and adaptation for people with visual disabilities aim to increase the friendliness of its reading, as well as its possible use from mobile phones. Respect for the Accessibility Guidelines for WEB Content (WCAG).

The web page is made up of:

Training content: grouped information, frequently asked questions document, self-assessment questionnaire, training videos and registration.
▪ Sections for special groups: people with chronic illness or special situations, pregnancy and lactation, children and caregivers.
▪ Press room: included a press kit to download according to the communication objectives of each campaign, press releases, press clipping, elements of future campaigns, etc.
▪ Frequently Asked Questions: Frequently asked questions document prepared by the General Directorate of Care, Humanization and Socio-Health Care in collaboration with the General Directorate of Public Health and the Agency for Food Safety, Environmental Health and Consumption.
▪ How much do you know? Self-assessment form with 16 questions to verify the level of basic knowledge about SARS-CoV2.
▪ Specific campaigns: images about the different campaigns that were carried out.
▪ Secondary Education Institutes of Asturias: This section was created to house the campaign and the contest that was carried out for school adolescents in Asturias.

According to Google Analytics web service, during the study period (March 2021–January 2022), the web page received 7080 visits from 5842 users (1.20 sessions per user). On each visit, users viewed an average of 1.70 pages from the main web page. The bounce rate, that is, the percentage of visitors who left the web page without taking any action, was 76%. Accesses to the web page were highest immediately after its creation, with a maximum of 500 weekly visitors between April and May 2021, and especially in December 2021, the week before the Christmas period, with more than 1000 weekly entries.

Of all the users of the web page, the age group most represented was that of 25–34 years (33.5%), followed by the group of 18–24 (24.5%), the group of 35–44 (15.5%) and that of 45–54 years (12.5%). Finally, those over 55 years of age accounted for 11% of accesses. Regarding sex, the percentage of men (54.2%) was slightly higher than that of women (45.8%).

**Figure 5:**
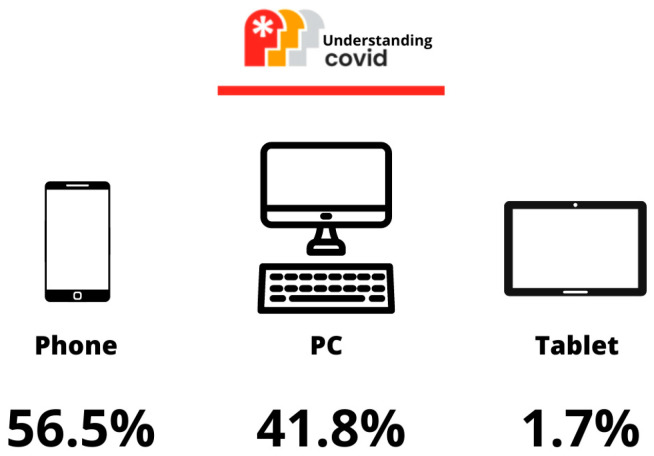
Device distribution.

In the analysis by country, accesses from Spain stood out (82.0%). Overall, Spanish-speaking countries accounted for more than 89.9% of accesses. Of the remaining percentage, the United States stood out with 2.08% and China with 1.90%. Finally, the devices used to access the website are shown in Figure 5.

#### 2.4.3. Actions for the Child and Adolescent Population

Following the WHO recommendations in times of pandemic fatigue, co-creation and participatory actions for the underage population were designed.

First, a creative contest for adolescents (from 12 to 18 years old) was run. In addition to the previously described adapted training sessions, a creative contest was held in collaboration with the Asturian Ministry of Education for the involvement of adolescents. First, an email was sent in April 2021 to all educational centers that provide secondary education, high school, and vocational training with an invitation and instructions for participation. The email contained a training video to be viewed in class with the students, which was definitively projected in 954 classrooms. The teachers encouraged debate and reflection on its content, and later, the students voluntarily created a creative product to compete in one of the following modalities: audiovisual, written, poster, and free creative. Campaign promoters received 111 creations of the four modalities, each one from a classroom of students between 12 and 16 years of age. In May 2021, the awards were delivered in a collaborative virtual ceremony organized by the Departments of Health and Education.

Second, a handwashing campaign called “*Bichos fuera*” (Bugs out!) was carried out in the Early Childhood and Primary Education Centers (from 3 to 11 years old). The teachers wrote the lyrics of a song in the Asturian language, designed the music, and devised the choreography and staging of the song “Bugs out!”. A video was recorded where the protagonists were the children (https://www.youtube.com/watch?v=vU1Kphiukdw, accessed on 1 February 2023). Classroom materials were also designed, such as cards and games. All the material was summarized in a guide for teachers (Supplementary Figure S1).

#### 2.4.4. Complementary Actions to Highly Exposed Workers/People

Some complementary actions were also carried out for specific activity sectors with high exposure to COVID-19 infection. For instance, a protocol for safe actions against COVID-19 was carried out during the celebration of Catholic masses indoors, with its corresponding posters (“Safe churches help us stop the COVID and reduce risks”). Moreover, a campaign called “First Quality Air” (Aire de Primera) was specifically designed for the hotel industry. Taking advantage of the Asturias tourist campaign “Asturias, Natural Paradise”, which promotes its pure and clean natural environment, posters of “First Quality Air” were prepared with COVID-19 protection measures to be used in hospitality and tourism establishments (Supplementary Figure S2).

#### 2.4.5. Other Dissemination Actions

To reinforce as well as to make room for consultation and reminders of the above elements, we also designed: (1) visual presentations with educational material adapted to different groups; (2) co-design of materials and recruitment mailings; (3) dissemination through social networks (Facebook, Twitter and WhatsApp) and mailing; and (4) a YouTube channel to host educational videos with simultaneous recording in sign language. The channel definitively hosted 10 training videos that had a total of 3,866 views.

## 3. Discussion

### 3.1. The “Understanding COVID” Strategy

The government of Asturias launched a public health campaign to improve the population response to the COVID-19 crisis and to fight against pandemic fatigue and infodemia. The main novelty of the “Understanding COVID” strategy consisted of specifically targeting a population selected on the basis of vulnerability criteria and identifying the topics on which they should be trained in. In addition, the training actions were delivered through a wide variety of methodologies tailor-made for recipients. For example, online training for all interested vulnerable citizens, posters for the productive sectors with the highest risk of transmission, pedagogical contests and educational games for schools, presence in social networks, etc. Definitively, 100 training actions were carried out for 21 subgroups of the vulnerable population, reaching more than 1000 individuals, but also students from almost 1000 classrooms and countless users from the hostelry industry and church sector.

A large number of graphic and audiovisual materials were developed that supported a positive and preventive discourse in the face of the COVID-19 pandemic. In addition, some of the materials disseminated in the school environment were co-created by children and adolescents since including them in the design was considered to increase acceptability.

One of the main challenges of the strategy was to reach as many vulnerable people of different ages as possible, as has been done in similar studies and interventions [15,16,17,18,19,20,21,22,23,24,25], but at the same time minimizing the technological gap that could leave someone behind, which is a common problem when trying to reach a vulnerable population using information technologies [9,26,27]. To do this, everyday technology tools already existing in homes, such as tablets and smartphones, were used, with no need for additional installation of complex programs. The design of the communication and dissemination strategy through digital technologies was in line with similar studies [28]. The good reception of the strategy “Understanding COVID” reinforced the choice of the method of dissemination and implementation, bringing this information accessible also to the population with hearing disabilities (with the support of professional translators in sign language) and visual (adaptation of audiovisual media).

Of all the actions of the “Understanding COVID” strategy, the ones that generated the most participation were those carried out in schools and in the catering sector since educational activities were added to the online training sessions in the centers and schools, and the distribution of posters occurred in restaurants. Gray et al. described the need to develop protection strategies within the school community and responded to an important need to provide information and support both to the teaching community and to families and students [29]. In our strategy, creativity and horizontal and ascending training were encouraged: from some students to others and from students to their parents. The information strategy in the hospitality sector through the “First Quality Air” campaign allowed commercial establishments to display posters with recommendations for the population, as well as to have a certificate accrediting the training received, thus promoting confidence and security among customers. As the restaurant industry is particularly sensitive to disasters, specific campaigns were run in some countries to encourage people to go out for lunch or dinner. Campaigns such as “Go to Eat” in Japan or “Eat Out to Help Out” in the United Kingdom applied discounts for dining in restaurants and simultaneously achieved an increase in sales and a rebound in cases [30,31].

The “Understanding COVID” strategy focused more on security and less on the economy because it was understood that by pursuing the first goal, it would achieve the second one. Finally, although the results referring to visits to the website are difficult to measure, it was relevant that the highest volume of unique visitors occurred two weeks before Christmas 2021, a time when the restrictions had been modified, and the population was looking for information to safely carry out trips, family reunions and other recreational activities. This increase in the number of hits to the page may reflect the confidence that the population has in seeking accurate, verified, accessible and adapted information, as was the objective of this strategy.

The “Understanding COVID” strategy contemplated some key elements that the scientific literature identifies for a campaign to be successful [32]. These include (1) messages that focus on the identity of the population, (2) the use of visual aids, and (3) the use of social networking features to encourage interaction. In addition, although it used online resources (web pages, webinars, social networks, etc.) to be consistent with the message of limiting physical and social contacts, other more appropriate resources were also used to reach the vulnerable population (posters, songs, etc.), which was somewhat less common in campaigns from other countries. In addition, it has been shown that the high penetration of mobile devices and technology in the younger population [33] opened a very interesting door to their inclusion in schools as a means to achieve early health literacy [34]. Additionally, parents of students can benefit from health literacy strategies from schools in collaboration with government health policies [17,35,36,37], as has been appreciated throughout this strategy.

### 3.2. Other Strategies and Campaigns

Most countries in the world disseminated information and prevention campaigns for COVID-19 through official statements and other mass media. In Spain, the national government developed four population campaigns exclusively on the internet in order to fight against the spread of the pandemic, reinforcing individual security measures and community action [38,39]. The campaigns were disseminated via Twitter, and the analysis of their design and implementation allowed some interesting conclusions to be drawn. Although the campaigns promoted the dissemination of health security measures, they did not serve to encourage debate and interaction between governments/public institutions and citizens [39]. In addition, the campaigns generated polar responses, with very positive visions that were faced with other very negative ones, which did not help to improve union and community action [38]. However, a similar campaign carried out in Italy through Facebook, the #I-am-engaged campaign, was built around a community perspective, with a participatory process that favored co-creation among peers. In addition, the campaign adopted a positive tone of voice by focusing on the promotion of good practices [40]. In these respects, the Italian campaign was similar to the “Understanding COVID” campaign, although the latter included a wide range of actions to be carried out beyond the digital world on the basis of trying to reach as many vulnerable people as possible.

Other campaigns carried out in various countries also tried to address the vulnerable population. For example, in the USA, an alliance of institutions launched a multifaceted national campaign whose objective was to increase confidence in vaccines and decrease misinformation within Hispanic communities. They successfully used social networks, webinars, radio and newsletters, with the participation of volunteers, key people for the Hispanic community and influencers [41]. In Maryland (USA), another regional campaign was developed through social networks and a web page to promote testing for COVID-19 and acceptance of the vaccine among Latinos with limited English proficiency [42]. Also, in the USA, campaigns were created on social networks to promote scientific information on the risks of COVID-19 in pregnancy and the benefits of vaccination, such as the “One Vax Two Lives” campaign in Seattle [43]. In Sydney, Australia, there were also efforts to engage culturally and linguistically diverse communities in the effective and appropriate public health response to COVID-19 [44]. A novel and rapid inter-agency campaign was established that included tailored public education and testing, the establishment of a local clinic, and inspections of local businesses to achieve a safe environment.

### 3.3. Lessons Learned and Limitations

An important lesson learned from the “Understand COVID” strategy was the importance of various public institutions working in a coordinated manner in pursuit of a common goal, something common to other similar campaigns [43,44]. It was also learned that in vulnerable populations, the public health response in crises must be adapted and react to their needs since, in these population groups, the information channels and conventional health messages are often insufficient. It was particularly interesting to see the acceptance of the campaign in the education sector, perhaps because teachers are very used to introducing transversal content into the academic curriculum, especially when the topic is linked to a problem in the real environment.

Another lesson provided by the implementation of this strategy is that in order to achieve successful health communication, the adoption of a participatory approach is essential where the stakeholders participate in the training and change process. In general, health communication based on evidence, culturally relevant and acceptable to the recipients is essential to educate and involve the population in situations that require a rapid and forceful response, either to educate about practical aspects or to combat the infodemic. The lessons learned in this strategy can be applied to other public health programs that seek to engage vulnerable communities.

The “Understanding COVID” strategy also presented some weaknesses.

First, the campaign was implemented in 2021, when pandemic fatigue was already becoming chronic. Bringing its launch back a few months might have been more successful in preventing fatigue. In addition, the execution deadlines for some activities to adapt to the environment where they were carried out (for example, actions in schools) and the evolution of the pandemic itself forced decisions to be made with little time for reflection.

Second, although most of the activities were always evidence-based and oriented towards infection prevention and management in a pandemic setting [45], other activities and groups, such as the promotion of physical activity [46], college students [47], and the ‘emotional well-being’ intervention [48], could have been taken more into account. On the contrary, it was decided not to focus solely on encouraging vaccination, as was done in many countries [49,50,51,52,53,54,55,56], since in Spain, the public response to the vaccine was very favorable, probably due to high confidence in the vaccination and in the health system [57].

Third, no data on the effectiveness of the campaign was obtained. This is a common limitation of public health campaigns, especially if they are launched under the pressure of an emergency. Evaluating the impact of public health strategies disseminated in an uncontrolled environment is a methodological challenge due to the many factors involved that can influence the results. In any case, at least one study based on surveys could have been carried out. It would have allowed us to know the impressions of people about the strategy. Although several opinion surveys were conducted, these were only used to tailor the strategy and not to explore the satisfaction of the participants in detail.

## 4. Conclusions

The “Understanding COVID” strategy was a public health campaign launched by the government of Asturias to improve the population’s response and adaptation to the COVID-19 crisis and to combat pandemic fatigue and infodemia. The main innovation of the campaign was to target a population selected on the basis of vulnerability criteria, whose voices were taken into account to identify training topics. Capacity building was achieved through a variety of tailor-made methodologies, such as online activities, posters for hotels and catering establishments, educational quizzes and games for schools, social media presence, etc. More than 100 training activities were conducted for 21 subgroups of the vulnerable population, reaching more than 1000 people, as well as students from almost 1000 classrooms and users of various hospitality establishments and vulnerable populations. The strategy faced the challenge of reaching as many vulnerable people of different ages as possible while minimizing the technological gap, which was addressed by using technologies accessible to the population, such as tablets and smartphones, that did not require large technological features. The “Understanding COVID” strategy was well-received and reinforced the choice of dissemination and implementation method, making the information inclusive for the deaf and visually impaired population (with the support of professional sign language translators and adaptations of audiovisual materials). The most participatory actions were those carried out in the school environment and in the hospitality sector, where educational activities were added to the online training sessions and posters were displayed in restaurants. The information campaign in the hospitality sector, “First Air Quality”, allowed commercial establishments to display posters with recommendations for the public and to have a certificate of the training received, promoting confidence and safety among customers.

Overall, the collaboration between different government agencies with the ultimate goal of reaching the population most vulnerable to the COVID-19 pandemic is possible if a coordinated strategy is developed that takes into account the citizens and their interests and adapts to their different needs.

## Figures and Tables

**Table 1:** Initial questionnaire for collecting information from citizens.

Question	Likert Scale *					
	0	1	2	3	4	5
Do you think it is necessary to receive training on how to carry out your work safely in times of coronavirus?	0%	2%	2%	2%	9%	84%
2. Do you think that receiving training from the Ministry of Health in the format of short videos that would reach your phone could be a good way to do so?	5%	3%	7%	9%	11%	65%
3. Do you think it is appropriate that this training includes information on how to act safely in the activities of your daily work (cleaning, cleaning, shopping,…)?	0%	2%	2%	3%	13%	80%
4. Do you think it appropriate that this training includes information on safe behavior rules during transfers between one home and another in your daily work?	0%	1%	4%	4%	12%	79%
5. Do you think it appropriate that this training includes information on how to disinfect your private car or that of your company that you use in your work transfers?	1%	2%	2%	4%	11%	80%
6. Does it seem appropriate to you that this training includes information on how to act when you feel a lot of stress or are overwhelmed at work?	0%	2%	1%	5%	10%	82%

* 0: “Strongly disagree” → 5: “Strongly agree”.

**Table 2:** Questionnaire for continuous collection of citizen information.

Questions	Options
Do you think that the training received is useful for you? (Usefulness)	6-item Likert scale 0 = not useful 5 = very useful
2. Is the training time adequate? (Time)	yes/no/don’t know
3. Has the training format been accessible to you? (Accessibility)	6-item Likert scale 0 = little accessible 5 = very accessible
4. General satisfaction with the training received. (Satisfaction)	11-item Likert scale 0 = no satisfaction 10 = very satisfied
5. Would you add any content to this training?	Open-ended question
6. Would you remove any content from this formation?	Open-ended question
7. Write any comment that you want to send us.	Open-ended question

**Table 3:** Percentage of agreement with usefulness, time and accessibility from the questionnaire for the continuous collection of information for citizens.

Type of Training	Usefulness (Likert)						Time			Accessibility (Likert)					
	0	1	2	3	4	5	Yes	No	Don’t Know	0	1	2	3	4	5
Families associations	0%	0%	0%	0%	22%	78%	97%	0%	3%	0%	0%	0%	2%	15%	83%
Secondary schools	0%	0%	0%	0%	14%	86%	100%	0%	0%	0%	0%	0%	0%	14%	86%
Drop by Drop	0%	0%	0%	4%	18%	77%	95%	1%	4%	0%	0%	0%	2%	15%	83%
First Quality Air	0%	0%	0%	3%	10%	88%	95%	5%	0%	0%	0%	0%	0%	20%	80%

**Table 4:** Percentage of agreement with satisfaction from the questionnaire for the continuous collection of information for citizens.

Type of Training	Satisfaction (Likert)										
	0	1	2	3	4	5	6	7	8	9	10
Families associations	0%	0%	0%	0%	0%	0%	2%	6%	9%	34%	49%
Secondary schools	0%	0%	0%	0%	0%	0%	0%	0%	4%	11%	86%
Drop by Drop	0%	0%	0%	0%	0%	0%	1%	2%	2%	5%	89%
First Quality Air	0%	0%	0%	0%	0%	0%	0%	5%	5%	5%	85%

**Table 5:** Target population reached in the “Understanding COVID” training.

Group /Environment	Specific Populations
Citizenship	Citizens in rural areas
	Citizens in urban areas
	Municipal technical professionals
	Ethnic minorities: Roma, Immigrants
	Citizens with impaired vision
	Citizens with impaired hearing
	Associations of people with mental health problems or addictions
	Non-professional caregivers
School environment	School-age students (6–12 years old)
	Secondary education and vocational training students (12–18 years)
	Families with school-age children ages 6–12 years
	Families Association of students aged 3 to 16 years according to educational levels of public and private centers and special education
Professional sector	Professionals from shelters
	Home caregiving professionals
	Non-professional caregivers
	Risk prevention services
	Hostelry
	Tourism
	Small business
	Religious groups

## Data Availability

Not applicable.

## Supplementary Materials

The following supporting information can be downloaded at: https://www.mdpi.com/article/10.3390/life13020589/s1, Figure S1: School material from the “*Bichos fuera*” (Bugs out!) campaign; Figure S2: “*Aire de Primera*”(First Quality Air) poster.
