# Supplementary material for: Understanding COVID: Collaborative Government Campaign for Citizen Digital Health Literacy in the COVID-19 Pandemic
Source: Life (Basel). 2023 Feb 20;13(2):589. doi: 10.3390/life13020589 (PMC9959963; doi:10.3390/life13020589)
Supplement: Supplementary file 1 [file life-13-00589-s001.zip › life-2161849-supplementary.pdf]

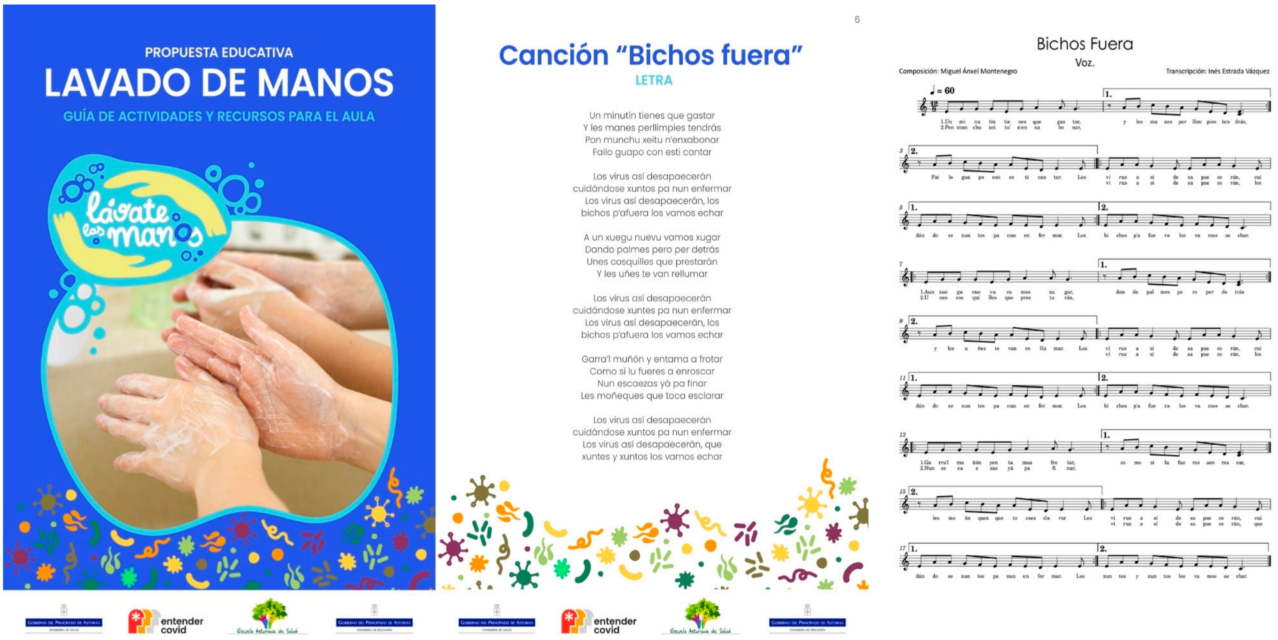

Suplementary Figure S1. School material from the "Bichos fuera" (Bugs out!) campaign

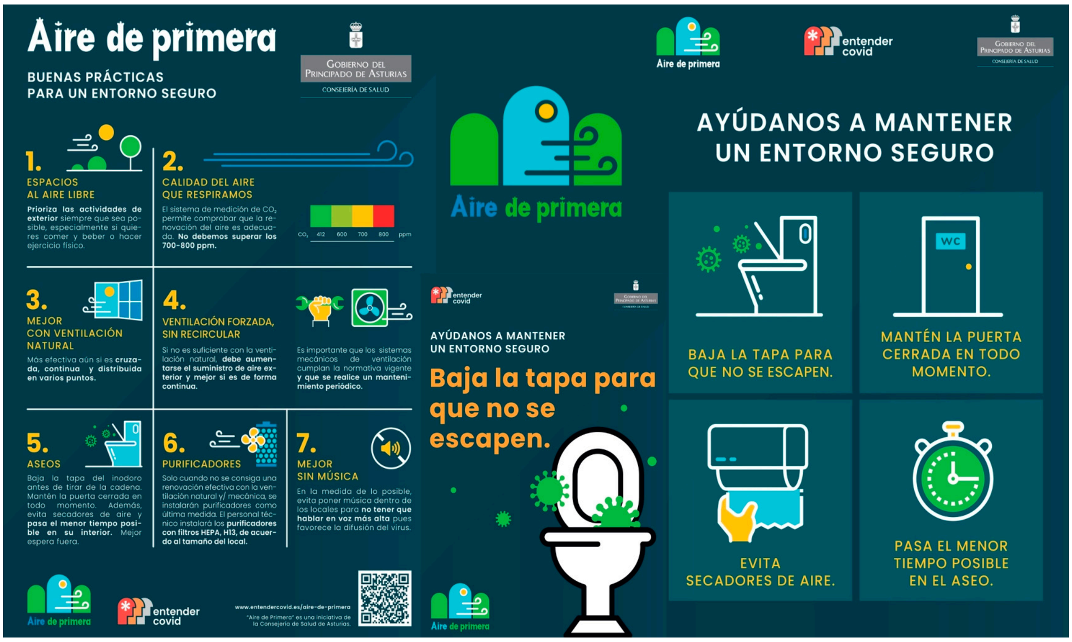

Suplementary Figure S2 "Aire de Primera"(First Quality Air) poster
